# Tactile feedback for relief of deafferentation pain using virtual reality system: a pilot study

**DOI:** 10.1186/s12984-016-0161-6

**Published:** 2016-06-28

**Authors:** Yuko Sano, Naoki Wake, Akimichi Ichinose, Michihiro Osumi, Reishi Oya, Masahiko Sumitani, Shin-ichiro Kumagaya, Yasuo Kuniyoshi

**Affiliations:** The Department of Mechano-Informatics, Graduate School of Information Science and Technology, the University of Tokyo, Eng. Bldg.2, 7-3-1, Hongo, Bunkyo-ku, Tokyo, Japan 113-8656; The Neurorehabilitation Research Center, Kio University, 4-2-2 Umaminaka, Kouryou-cho, Kitakatsuragi-gun, Nara, Japan 635-0832; The Anesthesiology and Pain Relief Center, the University of Tokyo Hospital, 7-3-1, Hongo, Bunkyo-ku, Tokyo, Japan 113-8656; The Research Center for Advanced Science and Technology, the University of Tokyo, 4-6-1 Komaba, Meguro-ku, Tokyo, Japan 153-8904

**Keywords:** Deafferentation pain, Phantom limb, Virtual reality, Tactile feedback, Bilaterality, Brachial plexus avulsion, Arm amputation

## Abstract

**Background:**

Previous studies have tried to relieve deafferentation pain (DP) by using virtual reality rehabilitation systems. However, the effectiveness of multimodal sensory feedback was not validated. The objective of this study is to relieve DP by neurorehabilitation using a virtual reality system with multimodal sensory feedback and to validate the efficacy of tactile feedback on immediate pain reduction.

**Methods:**

We have developed a virtual reality rehabilitation system with multimodal sensory feedback and applied it to seven patients with DP caused by brachial plexus avulsion or arm amputation. The patients executed a reaching task using the virtual phantom limb manipulated by their real intact limb. The reaching task was conducted under two conditions: one with tactile feedback on the intact hand and one without. The pain intensity was evaluated through a questionnaire.

**Results:**

We found that the task with the tactile feedback reduced DP more (41.8 ± 19.8 %) than the task without the tactile feedback (28.2 ± 29.5 %), which was supported by a Wilcoxon signed-rank test result (*p* < 0.05).

**Conclusions:**

Overall, our findings indicate that the tactile feedback improves the immediate pain intensity through rehabilitation using our virtual reality system.

**Electronic supplementary material:**

The online version of this article (doi:10.1186/s12984-016-0161-6) contains supplementary material, which is available to authorized users.

## Background

Phantom limb pain [1] is a common neuropathic pain syndrome following amputation [2], occurring in up to 80 % of patients [3, 4]. It is differentiated from non-painful phantom phenomena or residual-limb pain [5]. Because equivalent pain is induced by deafferentation such as brachial plexus avulsion as well as by amputation [2], we hereafter refer to the pain induced by deafferentation including amputation as deafferentation pain (DP).

Conventional treatments for DP frequently fail. The maximum reduction rate of about 30 % has been reported from symptomatic treatments such as pharmacological intervention, surgical operation, local anesthesia, and psychological and neurostimulation methods [5].

Mirror visual feedback (MVF) therapy has been proposed for phantom limb pain treatment by Ramachandran et al. [6, 7]. In MVF therapy, a visual image of a patient’s intact limb is displayed in place of the missing limb using a mirror. MVF therapy can help an upper limb amputee feel as if his or her phantom limb is moving together with his or her intact limb, thereby relieving the phantom limb pain. Another study showed that MVF therapy has efficacy for DP caused by brachial plexus avulsion injury in addition to that by limb amputation [8].

There have been some reports of brain activity correlated with MVF therapy [1, 9]. Flor et al. found via neuromagnetic imaging that a intensity of phantom limb pain and the reorganization of the primary somatosensory cortex (S1) are correlated [10]. Further, after successive MVF therapy, DP was relieved with a reversed dysfunctional cortical reorganization in S1 [11]. Diers et al. showed that both S1 and primary motor cortex (M1) were activated during MVF therapy in a fMRI study [12].

The original MVF therapy does not necessarily have a sufficient therapeutic effect on all patients with DP. The main problem is that MVF can present only a virtual visual image of the affected limb, which is often insufficient in terms of reflecting the reality of patients’ experiences.

Considering this limitation of MVF therapy, several studies have tried to relieve DP by using virtual reality systems. Sato et al. showed that four out of five patients with complex regional pain syndrome, after executing a target-oriented motor control task in a virtual environment, experienced pain reduction of more than 50 % [13]. Desmond et al. developed an augmented reality system capable of presenting asymmetric finger movements and applied it to upper limb amputees, with results showing that it relieved DP in one of three patients [14]. Cole et al. developed a virtual reality system for patients with arm or leg amputation that detects and translates the patient’s stump into virtual limb movements, resulting in pain amelioration of 22–100 % in ten out of 14 amputees [15]. They also reported that a feeling of agency and sensation was essential for the pain reduction. Murray et al. developed an immersive virtual reality system including a head mounted display for relieving DP [16–19].

These four studies are insufficient in terms of multimodal sensory feedback such as auditory and tactile input for representing the reality of phantom limb movements. Such input is crucial for enhancing the patient’s task experience, e.g., interacting with virtual objects by the virtual limb. Actually, the sound of hands clapping in synchrony with an upper limb amputee’s movement during MVF therapy was shown to improve his DP [20]. Furthermore, the tactile feedback to both intact and affected limbs was shown to be beneficial for alleviating the phantom pain of arm amputees when performing their advanced MVF therapy [21]. Therefore, it is expected that MVF therapy using a virtual reality technique could also be improved by incorporating auditory and tactile feedback.

We have developed a multimodal virtual reality MVF system that strongly enhances the reality of the patient’s experiences by introducing interaction between the virtual arm and virtual objects with visual, auditory, and tactile feedback. The objectives of this research are to apply this system to patients with DP and to validate the efficacy of the tactile feedback on immediate pain relief. The analgesic potency is evaluated by subjective indices—specifically, a short-form McGill pain questionnaire and questionnaire for sense of reality—and motor coordination is evaluated using an objective index—specifically, a correlation between the joint angles of the intact and affected arms. Some patients with deafferentation can move their affected arm to a small extent during MVF therapy, even though they usually have difficulty in smoothly and voluntarily doing so.

## Methods

### Participants

Seven patients with DP caused by brachial plexus avulsion or arm amputation participated in our experiments. The patients with brachial plexus avulsion could barely move their affected arms smoothly or voluntarily in daily life, but in our experiments, they occasionally moved their affected arms slightly.

The inclusion criteria were as follows: (1) diagnosis of upper limb deafferentation pain ipsilateral to injury after brachial plexus avulsion or arm amputation (irrespective of its origin); (2) mean pain intensity in the past week of >4 on an 11-point numerical rating scale (where 0 stands for no pain and 10 for worst possible pain); (3) pain duration >3 months; and (4) age between 20 and 80 years. Patients with cognitive dysfunction were excluded.

All participants were outpatients at the Department of Pain and Palliative Medicine, the University of Tokyo Hospital. This study was approved by the Ethical Review Board of the Faculty of Medicine, the University of Tokyo, and was conducted in accordance with the regulations of the Ethical Review Board. We explained the content and purpose of this study to all participants and obtained their written consent prior to participation in the experiments.

### System

#### Overall view

**Fig. 1:**
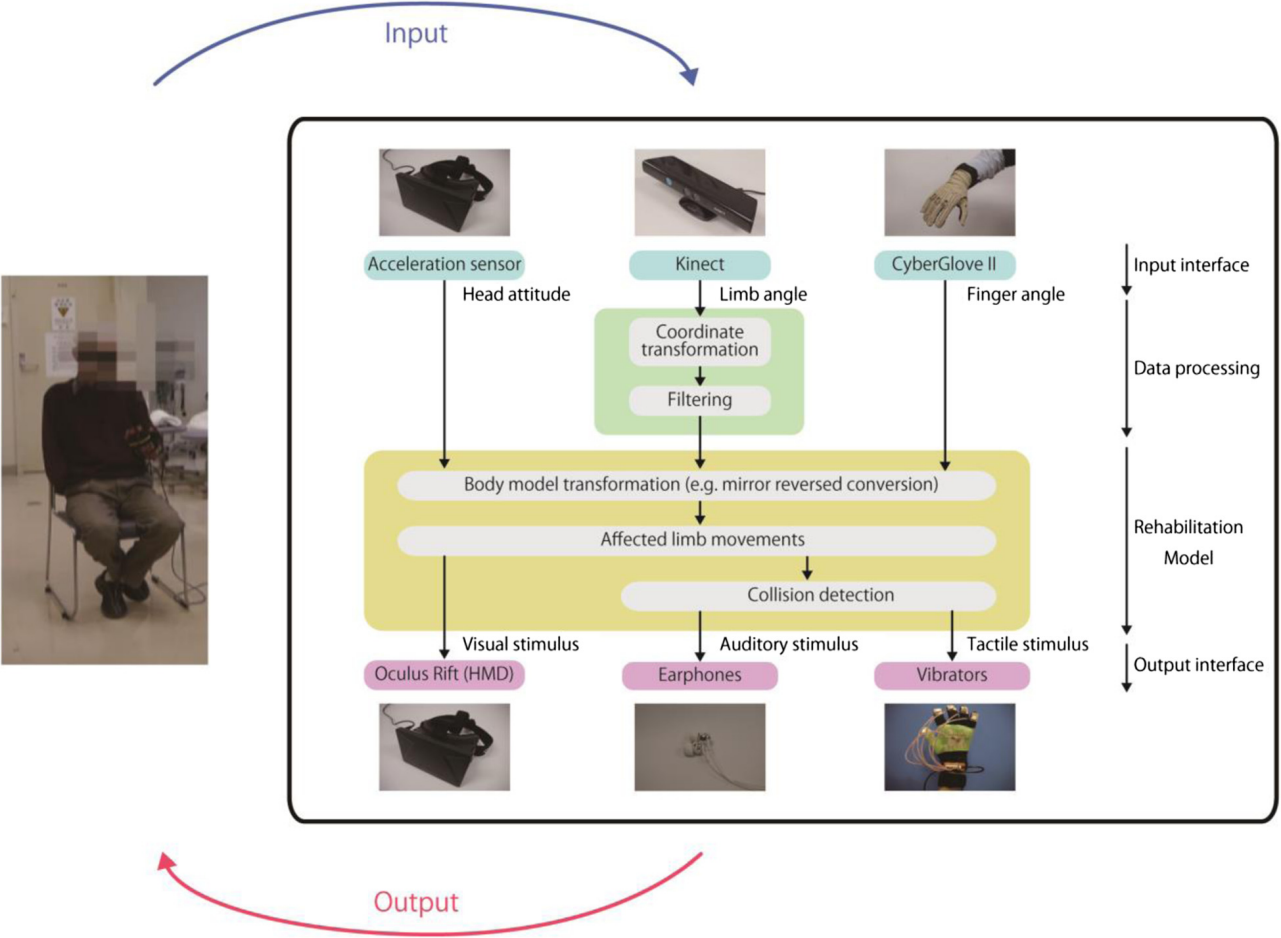
Overall view of our system. Movement of participants’ head, arms, and hand was detected by an acceleration sensor, Kinect, and a data glove, respectively. Movements of the intact arm and hand were converted symmetrically like a mirror-reversed image as the movement of the affected arm and hand. Collision detection between the affected arm and the target object was used to provide participants with an image of the arms and the target object, a collision sound, and a vibration on vibrating motors

An overall view of the system we have developed [22] is shown in Fig. 1. The movement of the participant’s arms was detected by a human motion detection system (Kinect™, Microsoft). The movement of a participant’s intact hand was detected by a data glove for detecting finger flexion (CyberGlove® II, CyberGlove Systems). The head angle was detected by an acceleration sensor attached to an immersive head mounted display (Oculus Rift™, Oculus VR). The movements of the intact arm and hand were transformed symmetrically as a mirror-reversed image to the movements of the affected arm and hand, displayed in the virtual environment. The participant watched the affected arm and the target object on the screen of the head mounted display.

The collision detection between the virtual affected arm and the virtual target object was calculated in real time. According to this calculation, an auditory stimulus (the collision sound) and a tactile stimulus (the vibration by the vibrating motor) were provided to the participant. Each participant executed reaching actions in which he/she was required to touch the target object with the virtual affected hand operated by the real intact arm/hand. When the affected hand reached the object, it disappeared with a collision sound and vibration.

### Details

#### Detection of arm movement

The movement of both the intact and affected arms was detected by Kinect ver. 1. Kinect detects movement of the human body by using RGB and depth images, making it advantageous in that it can detect movements without the optical markers typically required in motion capture systems. The position and orientation of each joint of the arms (shoulder, elbow, wrist, and hand) were recorded at 30 frames per second as quaternion values.

The joint values included noise due to errors in the estimation of body movement by Kinect. To reduce this noise, we applied an adaptive double exponential smoothing filter [23] implemented in Kinect for Windows SDK (Microsoft). The movement of fingers was detected by CyberGlove II. Kinect cannot detect fingers because they are often hidden behind each other, meaning that RGB and depth images have insufficient information pertaining to finger position. CyberGlove II, a glove with strain sensors, can detect the 18 angles of finger joints with an error of less than 1°.

### Mirror-reversed conversion

The position and orientation of arm joints and the angles of finger joints of the intact arm were transformed symmetrically with respect to the sagittal plane to those of the affected arm.

### Visual stimuli

**Fig. 2:**
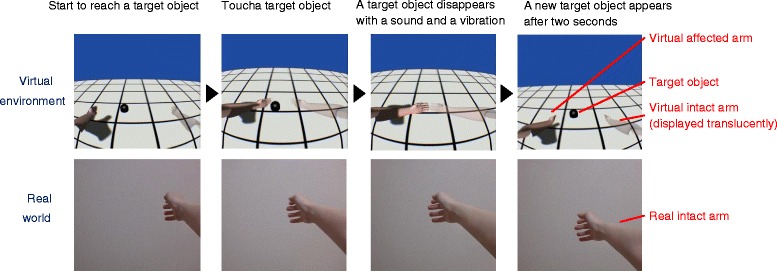
Reaching action. Participants tried to touch a target object with the virtual affected arm displayed on the left side in the virtual environment, which is operated by the real intact arm. The virtual intact arm moved mirror-symmetrically and was displayed translucently on the right side. Images in the virtual environment were changed in accordance with the head angle

Sample images of the affected arm and the target object as visual stimuli are shown in the upper half of Fig. 2. These images were displayed in a 3D world produced by Unity (Unity Technologies) on an Oculus screen. The intact arm was also displayed translucently so as to help participants imagine that both arms moved in a mirror-symmetrical manner.

Unity is a cross-platform game engine with an integrated development environment. Oculus is a head mounted display that displays an image stereoscopically and detects the angle of the participant’s head with an acceleration sensor. The head angle was filtered out with a Kalman filter [24] to reduce noise.

The head angle was input to Unity for controlling the image. In the virtual environment of Unity, two cameras were set 63.5 mm apart to match the average human interpupillary distance (default Unity setting). The 3D image was displayed stereoscopically using the two images taken by the cameras. The computer-graphic model of the arms was produced in a game engine Blender (Blender Foundation), which is especially suitable for 3D modeling. The models of the arms were the same size and color for all the participants. The target object was relocated to a random position on the virtual floor within reaching distance after every touch.

### Auditory stimulus

Auditory stimulus was provided to participants via earphones. When the affected hand reached the target object, a collision sound was played at equal volume for both ears.

### Tactile stimulus

Tactile stimulus was provided to participants by vibrating motors (4 F442, T.P.C. DC Motor) 12 mm in diameter, which are commonly embedded in cellular phones. The vibrating motors were placed on the five fingertips of each participant’s intact hand using a glove with a hook and loop fastener.

The vibrating motors were controlled by a motor driver (TA7291P, TOSHIBA) and a microcontroller (Arduino Uno™, Arduino). When the affected hand reached the target object, the vibrating motors were activated for 150 msec. This activation time was appropriate in terms of vibrotactile sensitivity [25].

Because the participants felt little or no sensory input on the affected hands, tactile stimulus was presented to the intact hands. This is expected to be effective with regard to intermanual referral of tactile sensations [1]. Actually, tactile feedback on the intact hand during MVF therapy elicited tactile sensation on the corresponding location of the phantom hand [7, 26].

### Task

We instructed the seven participants to move their real intact arms so as to reach the virtual target object with the virtual affected hand. We also asked each participant to imagine that both arms were moving symmetrically like MVF during the reaching task.

During the task, each participant executed the reaching actions shown in Fig. 2 repeatedly. The participant started to touch the target object with the virtual affected arm, as shown in the upper half of Fig. 2, which was operated by the contralateral real intact arm shown in the lower half of Fig. 2. When the participant succeeded in touching the target object, it disappeared with a collision sound and vibration. Then a new target object reappeared at a different position after a two-second break, and the participant began to try to touch it again. In the virtual environment, the intact arm was displayed translucently in a symmetrical position to the affected arm with respect to the sagittal plane.

At the beginning of each experimental session, each participant practiced the reaching action for a couple of minutes and then executed the reaching task for 5 min. Because the task required concentration, the duration of the task was kept short so as not to overtire the participants. The number of reaching actions during the task was recorded as described in the results section.

The task was executed under the following two conditions.

1. No tactile feedback condition
2. Tactile feedback condition

Each participant was evaluated one or more times under each condition (see Table 3). Several sessions under the same condition were conducted on different days at intervals of three weeks or more. In the case of different conditions examined on the same day, the experiments were conducted with breaks of at least five minutes in between so as to mitigate carry-over effects.

### Evaluation

The effect of the above-mentioned tasks was evaluated by subjective indices pertaining to pain intensity and sense of reality and by an objective index calculated from the trajectories of the intact and affected arms.

### Questionnaire for pain intensity

The intensity of DP was evaluated before and after the task using a short-form McGill pain questionnaire (translated into Japanese) consisting of 15 items on pain nature [27] (shown in Table 1). This questionnaire is used in clinical situations worldwide to quantify pain intensity. Each item was evaluated on a scale of 0 to 3 (0: none, 1: mild, 2: moderate, 3: severe). Participants answered orally so as not to disrupt the pain relief effect on the arms produced by the task. The sum of the score of all items, which was already reliably established as a common pain scale [27], was calculated (hereafter called “the McGill pain sum score”). The effect of pain relief was evaluated by the reduction rate (%) of the McGill pain sum score before and after the task, which is a commonly used index in clinical practice [28]. Here, a positive value in the reduction rate means a decrease of pain and a negative value means an increase.

**Table 1:** Short-form McGill pain questionnaire. Each question item was evaluated on a scale of 0 to 3. (0: none, 1: mild, 2: moderate, 3: severe)

No.	Question item
1	Throbbing
2	Shooting
3	Stabbing
4	Sharp
5	Cramping
6	Gnawing
7	Hot-burning
8	Aching
9	Heavy
10	Tender
11	Splitting
12	Tiring-exhausting
13	Sickening
14	Fearful
15	Punishing-cruel

### Questionnaire for sense of reality

After the task, the sense of reality of the affected arm in the virtual environment during the task was evaluated using the questionnaire in Table 2 (originally in Japanese). Q1 and Q2 relate to the ownership of the virtual affected arm. Q3 and Q4 relate to the agency of the virtual affected arm, not the real affected arm. These statements were inspired by the rubber hand experiment using a virtual reality system [29]. For each statement, the participants responded by choosing a score on a 7-point scale ranging from 0 for “none” to 6 for “extremely strong”. The sum of Q1 and Q2 was calculated as “the ownership score” and the sum of Q3 and Q4 was calculated as “the agency score”. The ownership and agency scores were not always consistent. For example, when the ownership score was low and the agency score was high, the participant would feel as if the virtual arm were a tool—i.e., that it was not his own arm but was under the control of his will.

**Table 2:** Questionnaire for sense of reality. Each statement was evaluated on a scale of 0 to 6 (0: none, 6: extremely strong)

Category	No.	Statement
Ownership	Q1	I felt as if the virtual arm was my real affected arm.
	Q2	I felt as if I was looking at my own arm.
Agency	Q3	I felt as if I could control the movements of the virtual hand.
	Q4	The virtual arm was obeying my will and I could make it move as I wanted to.

### Trajectory analysis of arm movement

**Fig. 3:**
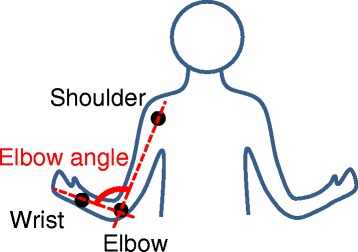
Elbow angle of participant. The elbow angle of a participant was defined as the angle between an elbow-shoulder vector and an elbow-wrist vector. Correlation between elbow angles of real intact and affected arms was calculated

The objective index of bilateral arm motor coordination, discussed in the background section, was calculated from the elbow angles (shown in Fig. 3) of the real affected and intact arms. The elbow angle was chosen because in the reaching task in our experiments, they changed much more dynamically than the other joints of the arms. The elbow angle is calculated from the joint positions measured by Kinect using

1 $$ {\theta}_{ea}=\frac{360}{2\pi } \arccos \kern0.6em \left(\frac{V_{es}\cdot {V}_{e\omega }}{\left|{V}_{es}\right|\left|{V}_{e\omega}\right|}\right), $$

where *θ*_*ea*_ is an elbow angle [deg], *V*_*es*_ is an elbow-shoulder vector, *V*_*eω*_ is an elbow-wrist vector, *V*_*es*_ ⋅ *V*_*eω*_ is an inner product of *V*_*es*_ and *V*_*eω*_, and | * | means a norm of the * vector. *θ*_*ea*_ was then put through a low-pass 1-Hz filter to eliminate noisy, quick motions. A cross-correlation between *θ*_*ea*_ of the intact arm and of the affected arm was calculated. The “similarity” of the intact and affected arms was defined as the absolute value of the cross-correlation at time lag = 0. We used the absolute value because a negative correlation, as well as positive one, indicates some coordination between the two arms.

### Statistical analysis

As for pain reduction rate, an average of the reduction rates was calculated for each participant under each condition. In order to test if the efficacy of the tactile FB was significant, the average reduction rates of all participants under the two conditions were subjected to a two-sided Wilcoxon signed-rank test [30] (between-group analysis). Additionally, the average reduction rates under each condition were subjected to the Wilcoxon two-sided signed-rank test to determine if they were significantly different from 0 (within-group analysis). The average ownership/agency scores of all participants under the two conditions were subjected to the two-sided Wilcoxon signed-rank test in the same manner as the pain reduction rate (between-group analysis). As for similarity of the elbow angles of the intact and affected arms, the average similarities of all participants under the two conditions were subjected to the two-sided Wilcoxon signed-rank test (between-group analysis). A Pearson’s correlation coefficient between the average ownership/agency scores and the pain reduction rates and that between the similarities of the elbow angles and the pain reduction rates were calculated. All the tests were calculated by MATLAB (R2014a).

## Results

### Participants

The details of the seven participating DP patients are given in Table 3. The cause of DP was brachial plexus avulsion (BPA) for six of them and arm amputation (AA) for one. None of the patients were in the acute stage, and the durations of their diseases were more than several years. The dominant hands were right for all participants before they had been affected. Four patients with complete BPA underwent an intercostal nerve transfer to restore upper extremity functions after the injury (Additional files 1, 2 and 3).

**Table 3:** Participants. Pain qualities indicate the top three items (or two items if several items tied for third place) in a short-form McGill pain questionnaire on the basis of average scores before tests. Non-painful sensations are added in parentheses if the participant reported them

No.	Sex	Age	Time since AA/BPA (years)	Affected hand	Disease	Completeness of BPA/Position of AA	Allodynia	Hypesthesia	Movement disorder	Intercostal nerve transfer	Pain qualities (non-painful sensation if any)	Number of tests	
												No tactile FB	Tactile FB
P1	Male	53	36	Left	BPA	Incomplete (C5,6)	+	+	+	No	Aching Throbbing Sharp (Numb)	1	3
P2	Male	49	26	Left	BPA	Complete	+	+	++	Yes	Throbbing Cramping Tiring-exhausting	1	2
P3	Male	54	20	Right	BPA	Complete	–	+	++	Yes	Cramping Gnawing Tiring-exhausting	2	5
P4	Male	47	14	Right	BPA	Complete	–	+	++	Yes	Cramping Tiring-exhausting,	1	4
P5	Male	75	9	Right	AA	Upper arm	(−)	(−)	(−)	–	Throbbing Shooting Cramping	1	3
P6	Male	46	21	Right	BPA	Incomplete (C5-8)	+	+	++	Yes	Hot-burning Tender Tiring-exhausting	2	3
P7	Male	56	6	Right	BPA	Complete	–	+	++	No	Throbbing Aching	1	2

*BPA* Brachial plexus avulsion, *AA* Arm amputation

### Result of deafferentation pain relief

**Fig. 4:**
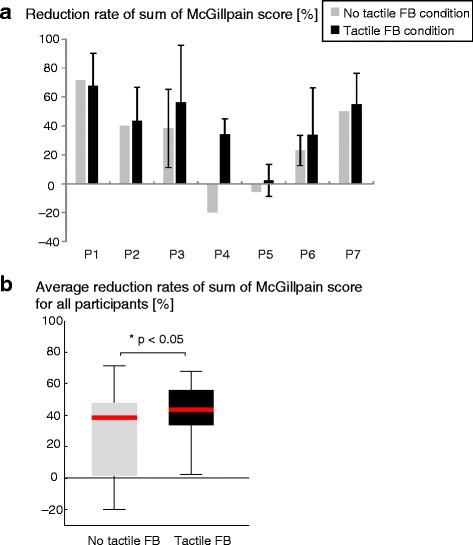
Reduction rate of McGill pain sum score under tactile feedback condition and no tactile feedback condition. **a** For all participants, reduction rates ranged from 2.2 to 67.8 % (average 41.8 ± standard deviation 19.8 %) under tactile FB condition and from −20.0 to 71.4 % (28.2 ± 29.5 %) under no tactile FB condition. **b** Average reduction rates under the two conditions were different with a significance probability *p* < 0.05 (*p* = 0.047). Negative values mean increase of pain. Error bars indicate standard deviations of reduction rates evaluated several times. No error bar means that a reduction rate was evaluated only once

**Fig. 5:**
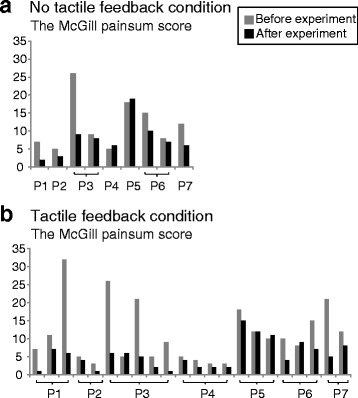
McGill pain sum scores before and after the experiment. **a** No tactile feedback condition. **b** Tactile feedback condition. Pain reduction rates shown in Fig. 4 were calculated from these scores. The upper limit of McGill pain sum scores is 45 and the lower limit is 0

The results of the reduction rate of the McGill pain sum score under both the tactile feedback (FB) and no tactile FB conditions are shown in Fig. 4 (a). The average reduction rates under the tactile FB condition ranged from 2.2 to 67.8 % (average 41.8 ± standard deviation 19.8 %) and that under the no tactile FB condition ranged from −20.0 to 71.4 % (28.2 ± 29.5 %), as shown in Fig. 4 (b). These average reduction rates under the two conditions were significantly different (*p* = 0.047 < 0.05). While the average of all reduction rates under the tactile FB condition was significantly different from 0 (*p* = 0.02 < 0.05), that under the no tactile FB condition was not significantly different from 0 (*p* = 0.078). Individually, for six out of seven participants (excluding P1), the reduction rate under the tactile FB condition was descriptively larger than that under the no tactile FB condition. All participants reported that the analgesic effect declined rapidly and lasted only a few minutes. The McGill pain sum scores before and after the experiment are shown in Fig. 5. During the task, each participant executed the reaching actions 62.4 ± 22.5 times on average (Additional files 4 and 5).

### Evaluation results of sense of reality

**Fig. 6:**
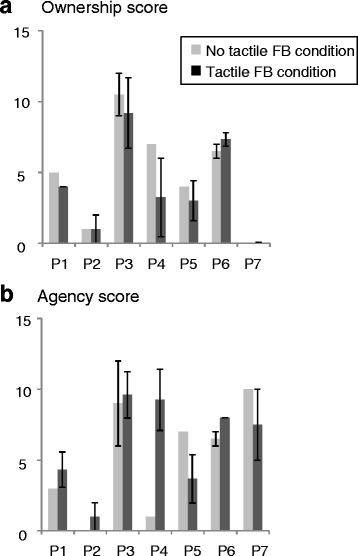
Reality scores. **a** Ownership score. **b** Agency score. Neither score was significantly different between the two conditions. However, the ownership score tended to be lower under the tactile FB condition than under the no tactile FB condition, while the agency score tended to be higher under the tactile FB condition than under the no tactile FB condition. Error bars indicate standard deviations of scores evaluated several times. No error bar means that a score was evaluated only once

The results of the reality scores (ownership and agency scores) are shown in Fig. 6. The average of the ownership scores under the tactile FB condition (4.0 ± 3.0) was lower than that under the no tactile FB condition (4.9 ± 3.3), even though the ownership scores under the two conditions were not significantly different (*p* = 0.13). Meanwhile, the average of the agency scores under the tactile FB condition (6.2 ± 3.0) was higher than that under the no tactile FB condition (5.2 ± 3.6), even though the agency scores under the two conditions were not significantly different as well (*p* = 0.69). The correlation coefficient between the average ownership/agency scores and the pain reduction rates under both of the conditions was −0.26 (*p* = 0.61)/0.22 (*p* = 0.67), respectively (Additional file 6).

### Trajectory analysis results of arm movement

**Fig. 7:**
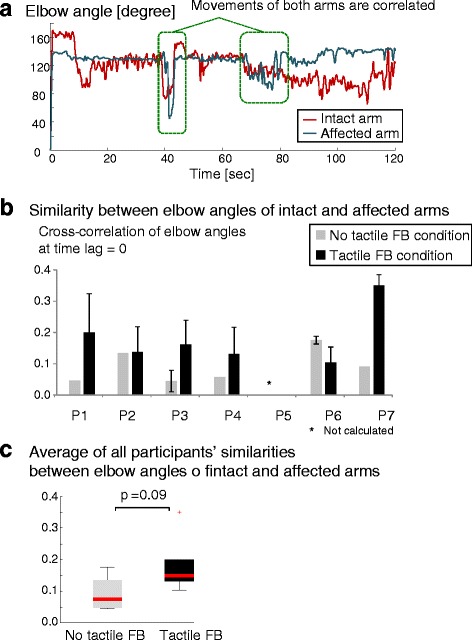
Elbow angle. **a** Waveforms of elbow angles of intact and affected arms. **b** Similarity between elbow angles of intact and affected arms. **c** Average of all participants’ similarities between elbow angles of intact and affected arms. In five out of six participants (excluding P6), similarities (cross-correlation function between intact arm and affected arm at time lag = 0) under tactile feedback condition were higher than those under no tactile feedback condition with *p* = 0.09. Similarity of P5 was not calculated due to lack of an affected arm (amputee). Error bars indicate standard deviations of similarities evaluated several times. No error bar means that a similarity was evaluated only once

The results of the trajectory analysis of arm movements are shown in Fig. 7. Figure 7 (a) depicts sample waveforms of the elbow angles of the intact and affected arms that were put through a low-pass filter (Additional file 7).

The similarities (excluding one arm amputee (P5)) are shown in Fig. 7 (b). The average of the similarities under the tactile FB condition (0.18 ± 0.08) was higher than that under the no tactile FB condition (0.09 ± 0.05) but not significantly (*p* = 0.09), as shown in Fig. 7 (c). For five out of six participants (excluding P6), the individual similarity under the tactile FB condition was descriptively higher than that under the no tactile FB condition. The correlation coefficient between the similarities and the pain reduction rates under both of the conditions was 0.30 (*p* = 0.57). Specifically, the correlation coefficient between the similarities and the pain reduction rates under the tactile FB condition was 0.55 (*p* = 0.25) while that under the no tactile FB condition was −0.086 (*p* = 0.87).

## Discussion

### Result of deafferentation pain relief

The result that DP was significantly more alleviated under the tactile FB condition than under the no tactile FB condition (*p* = 0.047 < 0.05) demonstrates that tactile FB might be effective for DP relief during neurorehabilitation using our virtual reality system. In addition, the result that the average pain reduction rate under the tactile FB condition was significantly higher than 0 (*p* = 0.02 < 0.05) shows that our virtual reality system would be effective for DP relief.

As for the mechanism behind this DP relief, we focus on the “sensorimotor incongruence theory” proposed by Blakemore et al. and McCabe et al. [31, 32]. According to their model, after limb amputation, the discrepancy between the predicted arm state based on “efference copy” (of the motor command signals) and the actual state based on sensory signals increases, and patients accordingly feel pain or the sensation of a new third limb [33, 34]. Although many studies agree with this theory [12, 35–38], its reliability remains highly controversial [39, 40]. As one of the several possibilities, we speculate that the tactile FB in the present study could help decrease the sensorimotor discrepancy, resulting in alleviation of DP.

In this study, the tactile stimulus was applied to the intact hands because the participants felt little or no sensory input on the affected hands. Nonetheless, the tactile stimulus was effective, probably because of intermanual referral of tactile sensations [1]. A relevant finding is that tactile feedback on the intact hand during MVF therapy elicited tactile sensation on the corresponding location of the phantom hand [6, 26].

The duration of the proposed task (5 min) was significantly short compared to the 30–90 min cited in previous studies [15, 17]. Considering these differences, our system might be advantageous in terms of relieving pain with less effort. However, the analgesic duration of the proposed task was only a few minutes. The relationship between the duration of the task and the analgesic duration or efficacy should be investigated in the future.

### Correlation between intact and affected arms

The bilateral arm movement similarity under the tactile FB condition tended to be higher than that under the no tactile FB condition (not significant, *p* = 0.09). This suggests the possibility that the correlation between the intact and affected arm movements might be an effective marker for evaluating the sensorimotor integration. Assuming that the sensorimotor incongruence theory is correct, the present result that the bilateral arm movement similarities and the pain reduction rates under the tactile FB condition were moderately correlated (*r* = 0.55, not significant, *p* = 0.25) might be consistent with this speculation. This speculation might also be consistent with the fact that five out of six participants with brachial plexus avulsion (excluding P7) had an incomplete injury or underwent an intercostal nerve transfer for restoring the functions of their affected limb.

The above speculation is relevant to the “convergence of sensory representation of bilateral hands” proposed by Sumitani et al. [41], which is the hypothesis that somatosensory information from bilateral hands would be convergent to one sensory representation in the hierarchical somatosensory processing. We extend their hypothesis, which is concerned with sensory information, to motor command: specifically, the convergent motor representation of bilateral arms. The correlation between the arms in the present study would be an outcome of the convergent motor representation of bilateral arms. One possible cortical area responsible for it may be the supplementary motor area [42]. Our instruction to move both arms symmetrically, as well as the translucent image of the affected arm displayed on the screen, probably helped to form the convergent motor representation of bilateral arms.

The higher correlation between the intact and affected arms with tactile FB suggests that motor commands to a phantom limb are reinforced as well as those to the real affected arm. In regard to the relationship between motor commands to a phantom limb and DP, Sumitani et al. proposed the hypothesis that voluntary movements of a phantom limb are associated with reduction of DP [43]. This hypothesis is consistent with a previous study indicating that DP could not be reduced by motor imagery without sensory input, and that visual feedback was essential [44], while another study showed that DP could be reduced by intensive 6-week training using only mental imagery [45]. Sumitani’s hypothesis is also supported by a study that found using a virtual reality system without a feeling of agency concerned with a phantom limb did not lead to any pain reduction, and that pain reduction always followed, but did not precede, the restoration of agency [15]. Another study using fMRI showed that pain reduction after successive MVF therapy was related to a decrease of activity in the inferior parietal cortex [11], which is a region that is activated when agency is interrupted [46]. It seems that the results reported in the present study—namely, that the agency score tended to be higher under the tactile FB condition than under the no tactile FB condition (not significant, *p* = 0.69)—might be consistent with these findings. Further study with a larger number of participants is required.

### Limitations of this study

The pain relief results were confined to immediate pain intensity, not long-term effectiveness. The relationship between the frequency and duration of the proposed task and the duration and intensity of the pain relief should be explored in detail in the future.

The present study was preliminary to the extent that only seven patients were evaluated and the number of tests was low. Previous studies have also presented preliminary experiments evaluating only 3–7 patients with pain in their arms [27–29, 31]. In the future, statistical significance by evaluating large numbers of patients should be demonstrated in a randomized controlled trial so as to properly confirm the efficacy of our proposed system.

The tactile FB in the present study was provided on the intact hand. In light of our finding that the tactile FB was effective for DP relief, we speculate that this tactile FB would be effective with regard to intermanual referral of tactile sensations [1]. However, if this speculation was not correct, new sensory incongruence between the affected hand and the intact hand could be induced. There is a possibility that the results reported here—namely, that the ownership score tended to be lower under the tactile FB condition than under the no tactile FB condition (not significant, *p* = 0.13)—might be consistent with this speculation. Thus, a more suitable body position to which tactile FB is provided should be investigated in future work.

The present results could possibly include a placebo effect and a distraction effect. Cole et al. have also suggested that a placebo effect may be present in rehabilitation using a virtual reality system [29]. However, our comparison of the two conditions was not influenced by the placebo effect because both conditions might include the placebo effect, thus cancelling it out. On the other hand, a distraction effect concerned with tactile perception could be induced only under the tactile FB condition. It is therefore possible that a distraction effect might have influenced the present results.

We conducted fewer experiments under the no tactile feedback condition than under the tactile feedback condition. Depending on the convenience of the participants, the experiments could not be performed several times. Previous studies have already shown that virtual reality MVF without tactile feedback was effective [27–30], so we gave priority to evaluating the tactile feedback condition in order to confirm its effectiveness for certain. Furthermore, the no tactile feedback condition tended to be conducted only at the beginning of the day, but not always (four out of seven participants). This experimental condition might have had an influence on the pain relief effect. However, because the pain relief effect last for only a few minutes, as mentioned in the results section, it is unlikely that the present result was influenced by that.

## Conclusions

In the present study, we applied a virtual reality system with multimodal sensory feedback to patients with deafferentation pain (DP) and validated the efficacy of the tactile feedback on immediate pain relief.

Seven patients with DP caused by brachial plexus avulsion or arm amputation executed a reaching task using a virtual affected limb operated by a real intact limb. The experiments were conducted under two conditions: one with tactile feedback on the intact hand and one without. The intensity of DP was evaluated after the experiments by using a questionnaire.

Results showed that the sum of the McGill pain score decreased from 41.8 ± 19.8 % after the task under the tactile feedback condition and decreased from 28.2 ± 29.5 % after the task under the no tactile feedback condition. These reduction rates under the two conditions were different with a significance probability *p* < 0.05. This finding indicates that tactile feedback strengthens the pain reduction effect of the task in the virtual reality system.

## Abbreviations

AA, arm amputation; BPA, brachial plexus avulsion; DP, deafferentation pain; FB, feedback; MVF, mirror visual feedback; S1, primary somatosensory cortex

## Additional files

Additional file 1: Experiment Dates. (XLSX 11 kb) Additional file 2: Video of virtual reality rehabilitation (participant). (WMV 11255 kb) Additional file 3: Video of virtual reality rehabilitation (screen). (WMV 2981 kb) Additional file 4: Reduction rates of McGill pain sum score. (XLSX 60 kb) Additional file 5: Number of reaching actions. (XLSX 11 kb) Additional file 6: Scores of sense of reality. (XLSX 28 kb) Additional file 7: Similarities of bilateral arm trajectory. (XLSX 14 kb)
